# Hippocampal neural stem cells are more susceptible to the neurotoxin BMAA than primary neurons: effects on apoptosis, cellular differentiation, neurite outgrowth, and DNA methylation

**DOI:** 10.1038/s41419-020-03093-6

**Published:** 2020-10-24

**Authors:** Paula Pierozan, Daiane Cattani, Oskar Karlsson

**Affiliations:** Science for Life Laboratory, Department of Environmental Science, Stockholm University, 114 18 Stockholm, Sweden

**Keywords:** Neuroscience, Stem cells, Neurological disorders

## Abstract

Developmental exposure to the environmental neurotoxin β-N-methylamino-l-alanine (BMAA), a proposed risk factor for neurodegenerative disease, can induce long-term cognitive impairments and neurodegeneration in rats. While rodent studies have demonstrated a low transfer of BMAA to the adult brain, this toxin is capable to cross the placental barrier and accumulate in the fetal brain. Here, we investigated the differential susceptibility of primary neuronal cells and neural stem cells from fetal rat hippocampus to BMAA toxicity. Exposure to 250 µM BMAA induced cell death in neural stem cells through caspase-independent apoptosis, while the proliferation of primary neurons was reduced only at 3 mM BMAA. At the lowest concentrations tested (50 and 100 µM), BMAA disrupted neural stem cell differentiation and impaired neurite development in neural stem cell-derived neurons (e.g., reduced neurite length, the number of processes and branches per cell). BMAA induced no alterations of the neurite outgrowth in primary neurons. This demonstrates that neural stem cells are more susceptible to BMAA exposure than primary neurons. Importantly, the changes induced by BMAA in neural stem cells were mitotically inherited to daughter cells. The persistent nature of the BMAA-induced effects may be related to epigenetic alterations that interfere with the neural stem cell programming, as BMAA exposure reduced the global DNA methylation in the cells. These findings provide mechanistic understanding of how early-life exposure to BMAA may lead to adverse long-term consequences, and potentially predispose for neurodevelopmental disorders or neurodegenerative disease later in life.

## Introduction

The environmental toxin β-N-methylamino-l-alanine (BMAA) is proposed as a risk factor for neurodegenerative disease, in particular amyotrophic lateral sclerosis/parkinsonism-dementia complex (ALS/PDC)^1–3^. This non-proteinaceous amino acid is produced by a variety of cyanobacteria (blue-green algae) and two groups of microscopic algae, diatoms and dinoflagellates^4,5^. As cyanobacteria are extensively distributed in terrestrial and aquatic environments all over the world, and eutrophication of aquatic environments together with global warming are promoting a rapid increase of the algae bloom^6^, BMAA may be an emerging global hazard. Humans can, for example, be exposed to BMAA via drinking water, recreational water, spray-irrigated food, seafood or even through the air^7–9^. Recent studies have demonstrated that BMAA also can be transferred from mussel-based feed into chicken^10^ and accumulate in birds’ eggs^11^ indicating that human consumption of these products can be an additional source of BMAA exposure.

While experimental studies have demonstrated a poor transfer of the toxin into the adult brain^12,13^, and a low neurotoxic potential in adult rodents^14^, BMAA is able to cross the placental barrier, and the uptake in discrete brain regions is more efficient in rodent fetuses and neonates^15^. In addition, BMAA is secreted into the milk of lactating rodents and distributed to the brain of suckling pups^16,17^. The relatively high uptake of BMAA in the developing brain is correlated with biochemical and behavioral changes in neonatal and juvenile animals^15,18,19^. Neonatal exposure to BMAA can also cause cognitive impairments^20,21^, proteomic alterations, and progressive neurodegeneration, including neurofibrillary inclusions, in the hippocampus of adult rats^22–24^. Since the hippocampus is essential for learning and memory, more studies on the developmental effects of BMAA in this brain area are necessary.

During development, the central nervous system is generated from a small number of neural stem cells^25^, and cell division, migration, differentiation into neurons, astrocytes and oligodendrocytes, neurite outgrowth and synapse formation proceed in a well-ordered manner. Dysregulation of any of these vital processes due to either genetic causes or environmental exposures may lead to disabilities or disease later in life^26^. Brain development is regulated by epigenetic mechanisms such as DNA methylation, and early-life exposure to environmental contaminants may impair neural stem cells reprogramming through epigenetic alterations, which could result in long-term consequences in the adult brain^27^. Neural stem cell cultures are, therefore, an important tool for mechanistic studies in the field of developmental neurotoxicology^28^.

The aim of this study was to compare the susceptibility between hippocampal neural stem cells and primary neurons to BMAA toxicity. We examined the effects of BMAA exposure on cell proliferation, differentiation, neurite outgrowth, global DNA methylation, and investigated if the effects persist in the absence of the exposure, and are inherited from one cell generation to another.

## Material and methods

### Chemicals

β-N-methylamino-l-alanine hydrochloride (≥97% purity, CAS Number 16012-55-8), paraformaldehyde, 4´,6-diamidino-2-phenylindoledihydrochloride (DAPI), Triton X-100, propidium iodide (PI), DNAse-free RNAse A, 3-(4,5-dimethyl-2-yl)2,5-diphenyl-2H-tetrazolium bromide (MTT), and basic fibroblast growth factor (bFGF) were obtained from Sigma-Aldrich Co (St. Louis, MO, USA). Bovine serum, penicillin–streptomycin, Dulbecco’s phosphate-buffered saline (PBS), neurobasal medium, poliornithine, fibronectin, trypsin solution (0.05%), glutamine and B27 were obtained from Gibco (Invitrogen, Paisley, UK). The secondary antibodies Alexa fluor 555 anti-mouse IgG, 488 goat anti-rabbit IgG, 350 donkey anti-goat IgG, 647 goat anti-chicken IgG, the blocking agent (normal goat serum) and the annexin-PI kit were obtained from Molecular Probes (Invitrogen, Paisley, UK). The antibodies MAP, β III-tubulin anti-rabbit, glial fibrillary acidic protein (GFAP) anti-mouse, nestin anti-rabbit, anti-5-methylcytosine (5-mc) and the secondary antibody HRP-conjugated goat anti-rabbit and anti-mouse were obtained from Abcam (Cambridge, UK). Apoptosis-inducing factor (AIF), caspase-3, cleaved caspase 3 (5A1E), caspase 12, and cytochrome c (136F3) were obtained from Cell Signaling (Boston, MA, USA). The antibody oligo4 was obtained from Chemicon (Temecula, CA, USA). The 5-methylcytosine and cytosine DNA standards were obtained from Zymo Research (Irvine, CA, USA).

### Animals and housing

Pregnant outbred Wistar rats, obtained from Charles River (Sulzfeld, Germany), were housed alone in Macrolon cages (59 × 38 × 20 cm) containing wood-chip bedding and nesting material. The dams were maintained on standard pellet food and water ad libitum. The animals were housed in a temperature and humidity-controlled environment on a 12-h light/dark cycle. All experiments were performed according to protocols approved by the Regional Animal Ethical Committee and following the Swedish Legislation on Animal experimentation (Animal welfare act SFS1998:56) and the European Union Directive on the Protection of Animals Used for Scientific Purposes (2010/63/EU).

### Cell culture and BMAA exposure

Pregnant rats were euthanized by decapitation^29,30^ and primary neuronal cell cultures were prepared from fetal hippocampus at embryonic day 18 as previously described^31^. In brief, a single-cell suspension was obtained by dissociating embryonic hippocampal cells in DMEM/F12 medium and 100,000 cells/cm^2^ were plated on polylysine-treated 96-well plates. Neuronal cultures were kept in neurobasal medium supplemented with 2 mM glutamine and B27 for up to 24 h. The medium was then replaced and the cells were incubated for 7 days in a humid incubator at 37 °C with 5% CO_2_. At 8 days in vitro, the culture medium was removed and cells were treated for 24 h with 50 µM to 3 mM BMAA dissolved in neurobasal medium and used for cell viability, proliferation and morphometric assays.

Neural stem cell cultures were prepared from fetal rat hippocampus at embryonic day 15 as previously described^31^. In brief, 40,000 cells/cm^2^ were plated in 75 cm^2^ flasks precoated with poly-l-ornithine and fibronectin and maintained in N2 medium enriched with 10 ng/ml bFGF to keep the cells in an undifferentiated state. After 3 days in culture, cells were passaged at low density (500 cells/cm^2^) on plates coated with poly-l-ornithine and fibronectin, in the presence of bFGF. One day after passaging, cells were treated with 50 µM to 3 mM BMAA for 24 h. After the exposure, medium without bFGF was added to promote spontaneous differentiation of the exposed neural stem cells for 24 h (cell viability and proliferation assays) or 7 days (morphometric and differentiation assays).

**Fig. 1:**
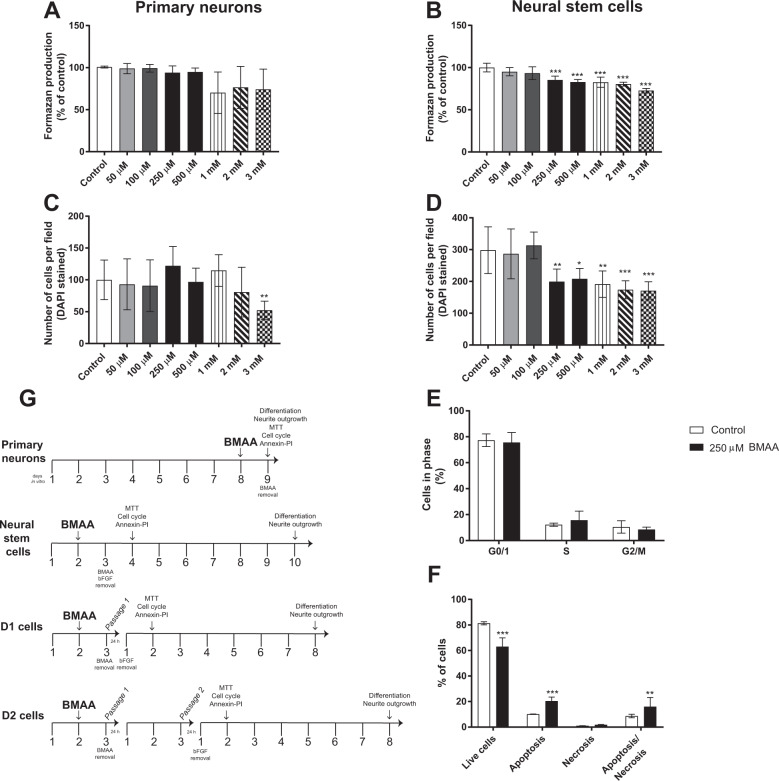
Effects of BMAA on cell viability and proliferation in hippocampal primary neurons and neural stem cells. Cell viability was determined using the MTT assay (**A**, **B**) and cell proliferation by counting the number of cells using DAPI staining (**C**, **D**) after treatment with 50 µM to 3 mM BMAA for 24 h. The cell cycle phases of neural stem cells treated with 250 µM were analyzed by flow cytometry (**E**), apoptotic and necrotic cells were assessed with the annexin V-PI assay (**F**). The experimental design used for investigating BMAA effects in hippocampal primary neurons and neural stem cells (**G**). Values represent mean ± SD from three independent experiments, each with six replicates. Statistically significant differences from control are indicated as follows: *p < 0.05, **p < 0.01 and ***p < 0.001 (one-way ANOVA followed by Tukey–Kramer test, or Student’s *t-*test when comparing only two groups).

The neural stem cells were also used to study mitotically heritable effects in daughter cells. The cells were exposed to non-cytotoxic concentrations of BMAA (50 or 100 µM) for 24 h and then passaged into their daughter cells (one passage for D1 and two passages for D2) and plated at low density (500 cells/cm^2^) (Fig. 1G). One day after passage one (D1) or passage two (D2), bFGF was removed and the cells were allowed to differentiate for 24 h (cell viability and proliferation assays) or 7 days (morphometric and differentiation assays). All experiments were performed using six replicates and repeated three times starting from the preparation of cell cultures from new animals.

### Cell viability and proliferation analysis

#### 3-(4,5-dimethylthiazol-2-yl)-2,5-diphenyltetrazolium bromide assay

MTT assay was performed as previously described^31^. Cell viability was measured after 24 h of BMAA exposure in primary neuronal and neural stem cells, and 24 h after the passage into the daughter cells. The formazan product generated was solubilized in dimethyl sulfoxide and measured at 490-630 using a SpectraMax i3 microplate reader (Molecular Devices, San Jose, CA, USA).

### Annexin V-PI labeling

The apoptotic/necrotic analysis was conducted by labeling cells with the Ca^2+^-dependent phosphatidylserine-binding protein annexin V and PI. Neural stem cells were detached from the culture plates by 0.05% trypsin-EDTA treatment, washed once with PBS and labeled by incubation with annexin V-FITC and PI at room temperature for 15 min in the dark, according to the manufacturer’s instruction. Stained cells were analyzed (10,000 events) on a Cytoflex flow cytometer (Beckman Coulter Ltd., Brea, CA, USA).

### Cell cycle analysis

Cells were processed for PI staining and flow cytometry as previously described^31^. Forward and light scatter data were collected in a linear mode, and fluorescence data from 10,000 cells per sample were collected in the FL3 channel on a linear scale. Side and forward light scatter parameters were used to identify the cell events and doublets cells were excluded using gating. Cells in different cell cycle phases were presented as a percentage of the total number of cells counted.

### Cell death signaling analysis and neural stem cells differentiation

#### Western blot

Proteins involved in the regulation of apoptosis were evaluated by western blot. Cells were lysed with Laemmli lysis buffer and the protein concentration was determined by Lowry assay^32^. An equal amount of protein was separated by sodium dodecyl sulfate-polyacrylamide gel electrophoresis (SDS-PAGE) on a 4-20% gel and transferred to nitrocellulose membranes (Mini Trans-Blot Electrophoretic Transfer Cell; Bio-Rad, Hercules, CA, USA). The blot was then incubated in a blocking solution (TBS; 500 mM NaCl, 20 mM Trizma, pH 7.8 with defatted dry milk), followed by washes with TBS and incubated overnight in TBS containing monoclonal antibodies (apoptosis-inducing factor (AIF), caspase 3, cleaved caspase 3 (5A1E), caspase 12 and cytochrome c (136F3)) diluted 1:5000. The blots were then washed with TBS and incubated for 1 h in TBS containing peroxidase-conjugated mouse anti-rabbit or anti-mouse IgG diluted 1:10000. The blot was developed with the chemiluminescence ECL kit (Bio-rad, Hercules, CA, USA) using a charge-coupled device (CCD) imager (Thermofisher, Rockford, IL, USA), and optical density was measured using the ImageJ Software. The results were normalized by the β-tubulin content and the protein levels were expressed as a percentage of control.

#### Immunocytochemistry and morphometric analysis

Immunocytochemistry was performed as previously described^31^. In brief, cells were plated at a density of 40,000 cells/cm^2^ on microscope glass coverslip precoated with poly-l-ornithine and fibronectin and treated with 50 or 100 µM BMAA for 24 h for cell differentiation and AIF analysis in neural stem cells. After that, BMAA and bFGF were removed and cells were allowed to differentiate for seven days. The cells were then fixed with 4% paraformaldehyde for 30 min and permeabilized/blocked with 1% BSA/0.1% Triton X-100 in PBS for 30 min at room temperature. Cells were incubated with AIF antibody (1:500) or with β III-tubulin (1:200), anti-GFAP (1:500), anti-nestin (1:1000) and anti-oligo4 antibodies (1:1000), at room temperature, followed by washes with PBS and incubation with secondary antibodies conjugated with Alexa 488 or 555 (1:1000) for 1 h. The nucleus was stained with DAPI (0.25 mg/ml) and the cells were examined in an Olympus IX70 inverted microscope (Olympus, Tokyo, Japan). The images were collected by a CCD camera with a 20x objective using constant intensity settings and exposure time for all samples. Semiquantitative analyses of differentiated cells were conducted in five random microscopic fields and images were analyzed with the ImageJ software (Sound Vision) after the digital acquisition. Negative controls were performed by omitting the primary antibody.

For morphometric analysis, primary neurons and neural stem cells were plated at a density of 40,000/cm^2^ on 96-well plates, and treated with 50 µM to 3 mM BMAA for 24 h. After that, cells were stained with β III-tubulin and MAP2 antibodies as described above. Mitotically inherited effects on the neurite outgrowth were analyzed in daughter cells of neural stem cells exposed to 50 or 100 µM BMAA. Images were collected with a 10x objective in an ImageXpress Micro XLS Widefield High-Content Analysis System (Molecular Devices, Sunnyvale, CA, USA). Nine fields per well were automatically analyzed with the MetaXpress Software after digital acquisition using the Neurite outgrowth application module, based on β III-tubulin staining.

#### Flow cytometry

To further study effects on cell differentiation, neural stem cells exposed to 50 or 100 µM and their daughter cells were fixed in 4% paraformaldehyde, permeabilized and blocked with 0.1% Triton X-100 and 1% BSA in PBS for 15 min. Then the cells were incubated with primary antibodies (anti-β III-tubulin, anti-GFAP, anti-nestin and anti-oligo4) at 4 °C overnight. After that, cells were stained with secondary antibodies (anti-rabbit Alexa 488, anti-goat Alexa 350, anti-chicken Alexa 647 and anti-mouse Alexa 555) for 60 min at room temperature. Samples were analyzed using a Cytoflex flow cytometer (Beckman Coulter Ltd., Brea, CA, USA). The quadrants to determine the negative and positive areas were placed on unstained and single stained samples. Forward and side light scatter gates were used to exclude cell aggregates and small debris. The number of cells in each quadrant was computed and the proportion of cells stained with the antibodies was calculated.

### Global DNA methylation

DNA was extracted from neural stem cells treated with 100 µM BMAA using the AllPrep DNA/RNA micro kit (Qiagen, Germany). The concentration of DNA was measured using Nanodrop 2000 spectrophotometer (Thermo Scientific, USA). The concentration of 5-methylcytosine was quantified by ELISA as previously described^33^. In brief, unmethylated DNA was used as negative control and the standard curve was prepared using methylated DNA. For each sample, 100 ng of DNA and PBS to reach the final volume of 100 µl were added to the PCR tubes. The DNA samples were denatured by heating at 98 °C for 5 min and then transferred immediately to ice for 10 min. The DNA samples were added to a 96-well microtiter plate, covered with aluminum foil and incubated at 37 °C for 1 h. After the incubation, the liquid content of the wells was discarded and the wells washed three times with 200 µl washing buffer (PBS with 0.2% Tween-20). The plates were blocked with a blocking buffer (Thermo Pierce, Rockford, IL, USA) at 37 °C for 30 min, and incubated overnight with anti-5-methylcytosine in PBS at 4 °C. The wells were then emptied and washed three times with washing buffer, and incubated with the HRP-conjugated secondary antibody for 30 min. After that, the wells were washed three times with washing buffer and 50 µl of 3,3′,5,5′-tetramethylbenzidine (TMB) substrate solution (Thermo Scientific Pierce, Waltham, MA, USA) was added into each well. The color reaction was terminated by the addition of 50 µl of stop solution (Thermo Scientific Pierce, Waltham, MA, USA). Optical density was measured at 450 nm using a SpectraMax i3 microplate reader (San Jose, CA, USA).

### Statistical analysis

The results are presented as mean ± standard deviation (SD) for each experimental group consisting of at least three individual cell cultures, each with five to six replicates. Differences compared with the control group were analyzed by one-analysis of variance (ANOVA) followed by Tukey–Kramer multiple tests, or by Student’s *t*-test when comparing only two groups (cell cycle and flow cytometry analysis) using Prism 7 (Graphpad Software, San Diego, CA, USA).

## Results

### Effects of BMAA exposure on cell viability and proliferation in primary neurons and neural stem cells

Initially, we examined the effects of 50 µM to 3 mM BMAA on cellular viability and proliferation in hippocampal primary neurons and neural stem cells. The results showed that BMAA exposure did not affect cell viability in primary neurons when measured with the MTT assay (Fig. 1A), and the cell number was decreased only at 3 mM BMAA (Fig. 1C). However, in the neural stem cells, BMAA induced a decrease in cell viability (Fig. 1B) and cell number (Fig. 1D) from 250 µM to 3 mM BMAA.

The lowest BMAA concentration that reduced the cell viability and cell number in the neural stem cells (250 µM) was then used to analyze the effects on cell cycle and cell death mechanisms by flow cytometry. While the results revealed no effects of BMAA exposure on the cell cycle (Fig. 1E), the Annexin V-PI assay demonstrated a decrease in the number of viable cells and an increase of cells in apoptosis and apoptosis/necrosis in the neural stem cells compared with the control group (Fig. 1F).

**Fig. 2:**
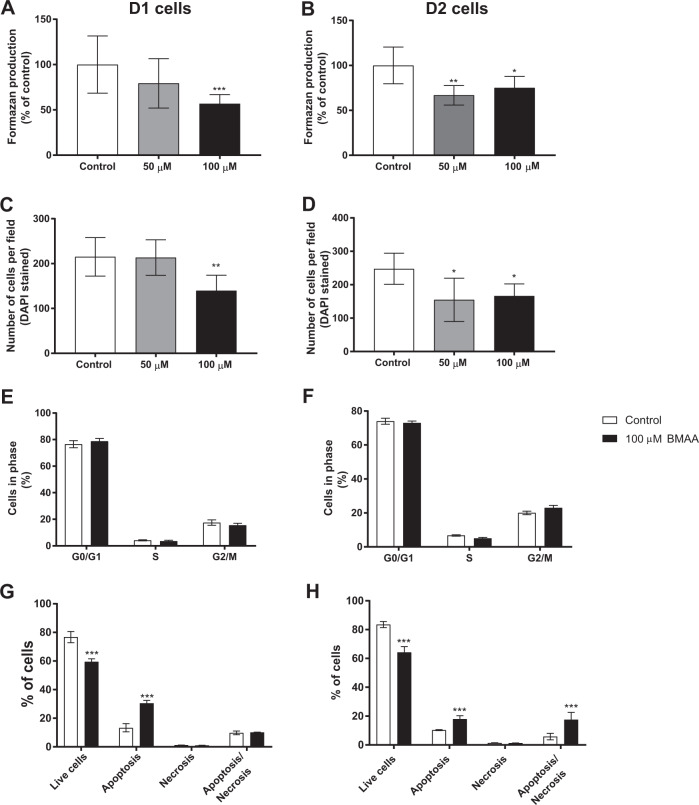
Effects of BMAA on neural stem cell viability and proliferation in daughter cells after one (D1) or two passages (D2). Cell viability was determined using the MTT assay (**A**, **B**) and proliferation by counting the number of cells using DAPI staining (**C**, **D**) in daughter cells of neural stem cells treated with 50 or 100 µM BMAA. The cell cycle phase was analyzed by flow cytometry (**E**, **F**), apoptotic and necrotic cells were assessed with the annexin V-PI assay (**G**, **H**) in daughter cells of neural stem cells exposed to 100 µM BMAA. Values represent mean ± SD from three independent experiments, each with six replicates. Statistically significant differences from control are indicated as follows: *p < 0.05, **p < 0.01 and ***p < 0.001 (one-way ANOVA followed by Tukey–Kramer test, or Student’s *t*-test when comparing only two groups).

Mitotically heritable effects of BMAA exposure on cell viability and proliferation were investigated in daughter cells of neural stem cells exposed to non-cytotoxic doses of BMAA (50 and 100 µM) (Fig. 1G). In contrast to the exposed cells, the results demonstrated a decreased cell viability in D1 (100 µM) and D2 (50 and 100 µM) cells compared with their respective control groups (Fig. 2A, B). This was confirmed by the cell counting (Fig. 2C, D) illustrating that the effects on cell viability and proliferation are shown in the daughter cells at even lower concentrations than in the neural stem cells actually exposed to BMAA. Cell cycle effects and cell death mechanisms were evaluated in the daughter cells of neural stem cells exposed to 100 µM BMAA. The results demonstrated no alteration in the cell cycle phases (Fig. 2E, F). To confirm that the decreased cell proliferation in the daughter cells is due to an increase in cell death, similar to the exposed neural stem cells, we performed annexin V-PI assay also in the daughter cells. The results showed an increase in apoptosis in D1 cells (Fig. 2G), and apoptosis and apoptosis/necrosis in D2 cells (Fig. 2H).

**Fig. 3:**
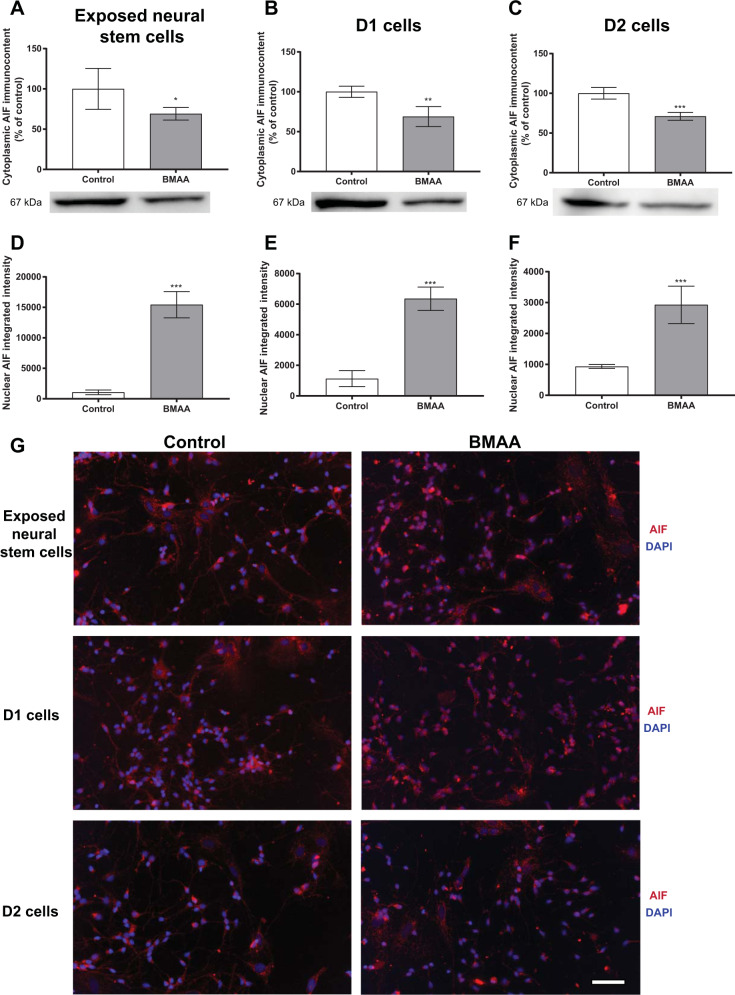
Involvement of the apoptosis-inducing factor (AIF) in cell death triggered by BMAA in neural stem cells. AIF levels were evaluated by western blot in cytoplasmic homogenates from neural stem cells exposed to 250 µM BMAA (**A**), or D1 (**B**) and D2 (**C**) daughter cells of neural stem cells exposed to 100 µM BMAA. β-tubulin was used as a loading control. Representative blots of three experiments are shown. Nuclear AIF levels were analyzed by immunocytochemistry using ImageJ (**D**–**F**). Representative images are shown (**G**). Values represent mean ± SD from three independent experiments. Statistically significant differences from control are indicated as follows: *p < 0.05, **p < 0.01 and ***p < 0.001 (Student’s *t-*test). Scale bar: 30 µm.

To better clarify the signaling pathways of cell death triggered by BMAA in neural stem cells, we studied proteins involved in mechanisms of apoptosis (cleaved caspase-3, caspase 12, cytochrome c and AIF) by western blot. The analysis was conducted in neural stem cells treated with 250 µM BMAA and in daughter cells of neural stem cells treated with 100 µM BMAA. The toxin induced no alterations in cytochrome c, cleaved caspase 3/caspase 3 and caspase 12 levels (Supplementary Fig.). However, the AIF levels in the cytoplasmic homogenate was decreased in the exposed neural stem cells (Fig. 3A) and in D1 and D2 cells (Fig. 3B, C). As AIF translocate from mitochondria to the nucleus when apoptosis is induced, we evaluated the effects of BMAA on AIF translocation through immunocytochemistry. The image analysis revealed an increased AIF translocation to the nuclei in the neural stem cells exposed to 250 µM BMAA (Fig. 3D) and in both D1 and D2 cells (Fig. 3E, F, respectively) derived from neural stem cells exposed to 100 µM BMAA. Representative images are shown in Fig. 3G.

### BMAA exposure reduces neural stem cell differentiation

**Fig. 4:**
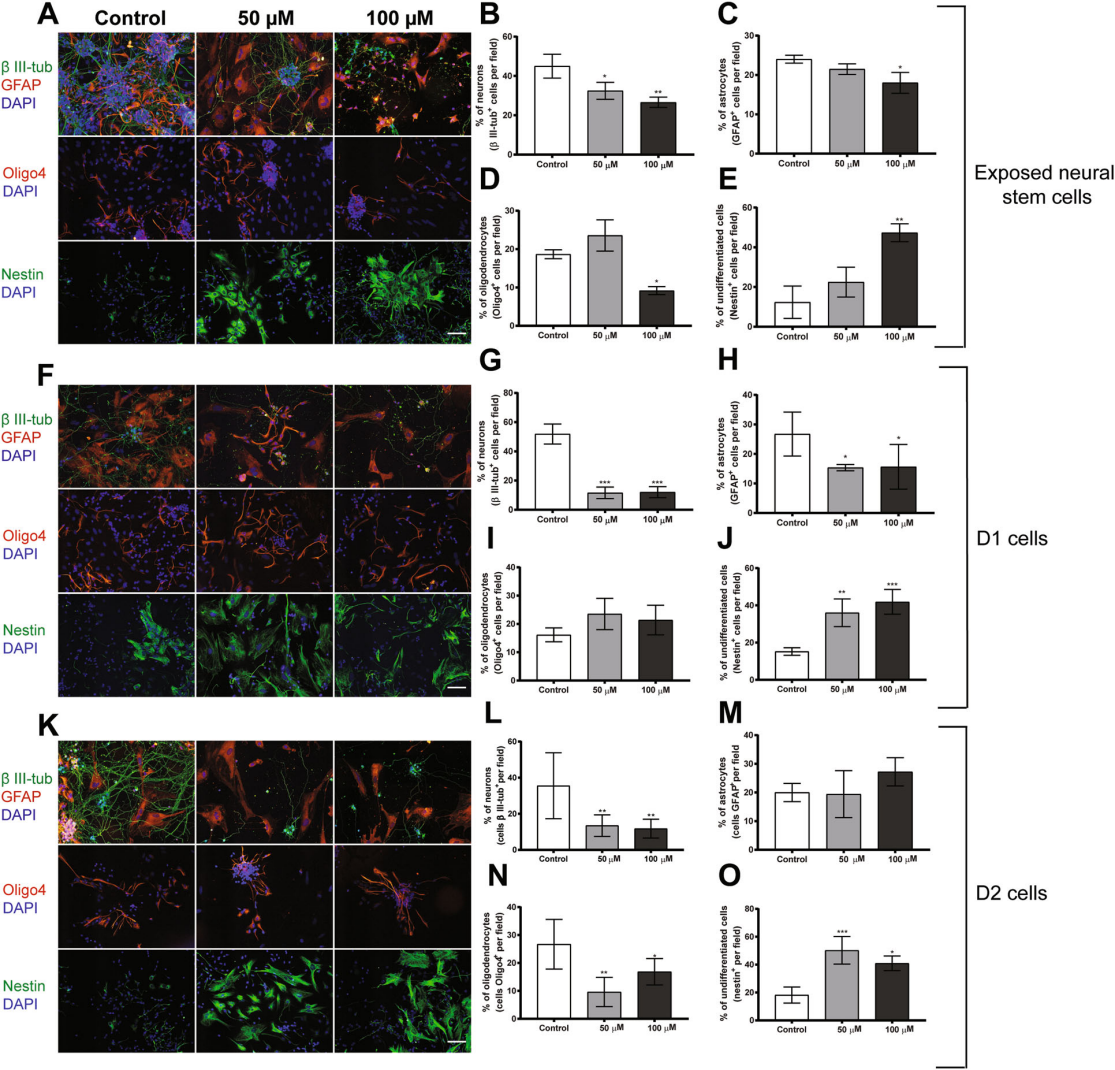
BMAA exposure to 50 or 100 µM alters the differentiation of neural stem cells and their daughter cells (D1 and D2). The cells were stained with the neuronal marker, β III-tubulin; astroglia marker, GFAP; the oligodendrocyte marker, Oligo4; and undifferentiated cells were marked with nestin. Nuclei were stained with DAPI. Representative images are shown (**A**, **F**, and **K**). Semiquantitative analyses of differentiated neurons (**B**, **G**, **L**), astrocytes (**C**, **H**, **M**), oligodendrocytes (**D**, **I**, **N**), and undifferentiated cells (**E**, **J**, **O**) were performed according to the material and methods section. Values represent mean ± SD from three independent experiments each with six replicates. Statistically significant differences from control are indicated as follows: *p < 0.05, **p < 0.01, and ***p < 0.001 (one-way ANOVA followed by the Tukey–Kramer test). Scale bar = 30 µm.

We examined the ability of BMAA to alter neural stem cell differentiation into neurons, astrocytes and oligodendrocytes in neural stem cells exposed to 50 or 100 µM BMAA and their daughter cells. Representative images of exposed cells are shown in Fig. 4A. Exposure to BMAA caused a significant decrease in the percentage of neural stem cell-derived neurons (Fig. 4B) at both concentrations, while only 100 µM BMAA caused a decrease in the percentage of astrocytes (Fig. 4C) and oligodendrocytes (Fig. 4D), as well as a significant increase in the percentage of undifferentiated cells (Fig. 4E). The effects on cell differentiation were also analyzed by flow cytometry, in neural stem cells treated with 100 µM BMAA and demonstrated a reduction in the percentage of neurons and oligodendrocytes, and an increase in the percentage of undifferentiated cells (Table 1).

**Table 1:** Effects on differentiation in neural stem cells exposed to 100 µM BMAA and their daughter cells (D1 and D2).

Cells	Control	BMAA
Exposed neural stem cells		
Neurons	43.38 ± 4.15	22.9 ± 1.92***
Astrocytes	24.38 ± 3.25	27.49 ± 13
Oligodendrocytes	10.24 ± 3.45	4.17 ± 1.91*
Undifferentiated cells	21.26 ± 4.94	46.27 ± 7.73*
D1 daughter cells		
Neurons	36.98 ± 8.88	17.74 ± 7*
Astrocytes	25.34 ± 3.97	11.31 ± 2.23*
Oligodendrocytes	13.84 ± 6.85	11.74 ± 2.5
Undifferentiated cells	22.83 ± 12	59.2 ± 8**
D2 daughter cells		
Neurons	40.73 ± 5.52	22.68 ± 3.48**
Astrocytes	22.67 ± 5.75	29.23 ± 16
Oligodendrocytes	25.28 ±	10 ± 2.06**
Undifferentiated cells	11.4 ± 5.9	40.02 ± 12*

Results are expressed as percentage for total events (10,000 events).

Statistically significant differences from control are indicated as follow: ***p < 0.001; **p < 0.01, and *p < 0.05 (Student’s *t-*test).

The mitotically inherited effects of BMAA on cell differentiation were investigated in the daughter cells after a multitude of cell divisions. Representative images of D1 and D2 cells are shown in Fig. 4F, K, respectively. The results revealed that the reduction of the neuron differentiation was persistent in D1 and D2 cells (Fig. 4G, L, respectively) for both concentrations, while the reduction in astrocyte differentiation only persisted in D1 cells (Fig. 4H, M). A reduction in oligodendrocytes was only observed in D2 cells (Fig. 4N). The percentage of undifferentiated cells were increased in both D1 and D2 cells (Fig. 4J, O, respectively). These immunocytochemistry results were confirmed by flow cytometry analysis (Table 1).

### Effects of BMAA on neuronal morphology

**Fig. 5:**
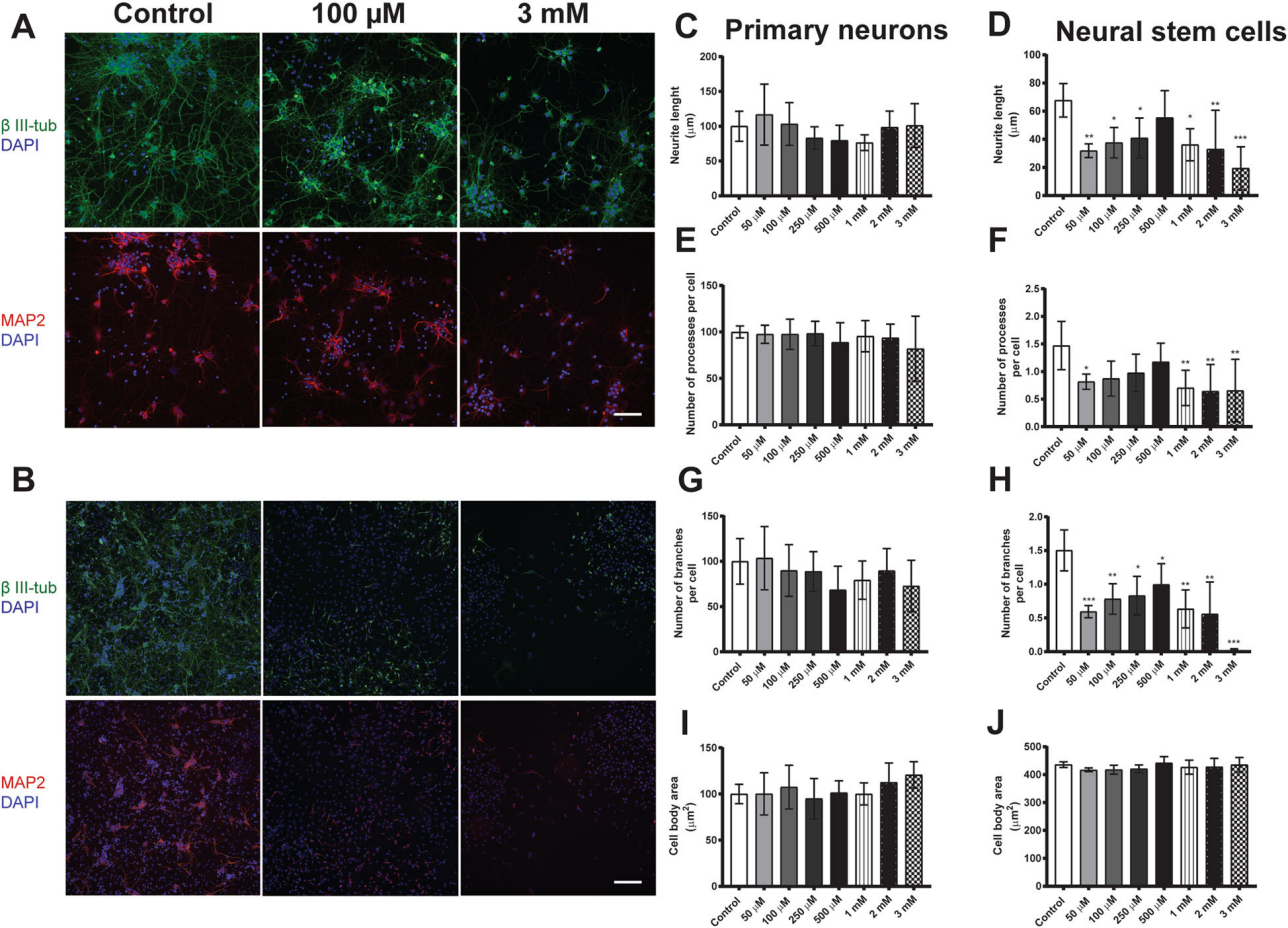
BMAA causes morphometric alterations in neurons derived from hippocampal neural stem cells but not in primary neurons. The effects on BMAA treatment with 50 µM to 3 mM BMAA was investigated directly after 24 h exposure of primary neurons, while the neural stem cells were allowed to differentiate for 7 days after the exposure. Representative images of cells immunostained with anti-β III-tubulin (green), anti-MAP2 (red), and DAPI (blue) are shown (Primary neurons, **A**; Exposed neural stem cells, **B**). Morphometric analysis was conducted using an ImageXpress Micro XLS Widefield HCA System (Molecular Devices, Sunnyvale CA, USA), where images were automatically captured and analyzed with the MetaXpress Software. Neurite length (**C**, **D**), the number of processes per cell (**E**, **F**), the number of branches per cell (**G**, **H**), and the cell body area (**I**, **J**) were determined. Values represent mean ± SD from three independent experiments, each with five to six replicates. Statistically significant differences from control are indicated as follows: *p < 0.05, **p < 0.01, and ***p < 0.001 (one-way ANOVA followed by Tukey–Kramer test). Scale bar = 50 µm.

Immunocytochemical staining with anti-β III-tubulin and anti-MAP2 antibodies were performed to analyze morphological parameters of primary neurons and neurons derived from neural stem cells treated with 50 µM to 3 mM BMAA. Representative images are shown in Fig. 5A, B for 100 µM and 3 mM BMAA, respectively.

BMAA exposure caused no morphological alterations in primary neurons at any of the concentrations (Fig. 5C, E, G, and I). In contrast, neurons derived from neural stem cells treated with 50 µM to 3 mM BMAA demonstrated a reduction in the neurite length (Fig. 5D), the number of processes per cell (Fig. 5F) and the number of branches per cell (Fig. 5H). The cell body area was not affected (Fig. 5J).

**Fig. 6:**
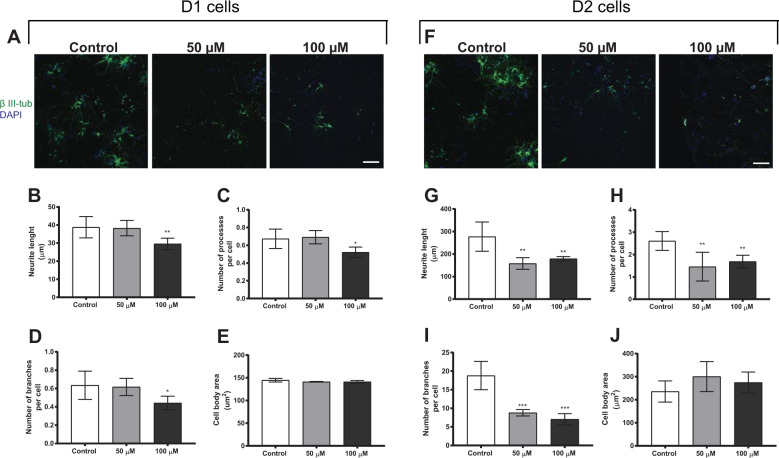
Morphometric alterations in neurons derived from daughter cells (D1 and D2) of neural stem cells treated with 50 or 100 µM BMAA. Representative images of cells immunostained with anti-β III-tubulin (green) and DAPI (blue) (D1 cells, **A**; D2 cells, **F**). Morphometric analysis was conducted using an ImageXpress Micro XLS Widefield HCA System (Molecular Devices, Sunnyvale, CA, USA), where images were automatically captured and analyzed with the MetaXpress Software. Neurite length (**B**, **G**), the number of processes per cell (**C**, **H**), the number of branches per cell (**D**, **I**), and the cell body area (**E**, **J**) were determined. Values represent mean ± SD from three independent experiments, each with six replicates. Statistically significant differences from control are indicated as follows: *p < 0.05, **p < 0.01 and ***p < 0.001 (one-way ANOVA followed by Tukey–Kramer test). Scale bar = 50 µm.

Morphological alterations were also investigated in neurons derived from daughter cells of neural stem cells treated with 50 or 100 µM BMAA, and most of the effects on neuronal morphology persisted in D1 and D2 cells. Representative images of D1 and D2 cells are shown in Fig. 6A and F, respectively. The results revealed a decrease in the neurite length (Fig. 6B), the number of processes (Fig. 6C) and the number of branches per cell (Fig. 6D) in D1 daughter cells of neural stem cells exposed to 100 µM of BMAA, while both 50 and 100 µM BMAA decreased the neurite length (Fig. 6G), the number of processes (Fig. 6H) and the number of branches per cell (Fig. 6I) in D2 cells. No alterations were observed in the cell body area of D1 or D2 cells (Fig. 6E, J, respectively).

### BMAA alters global DNA methylation in hippocampal neural stem cells

**Fig. 7:**
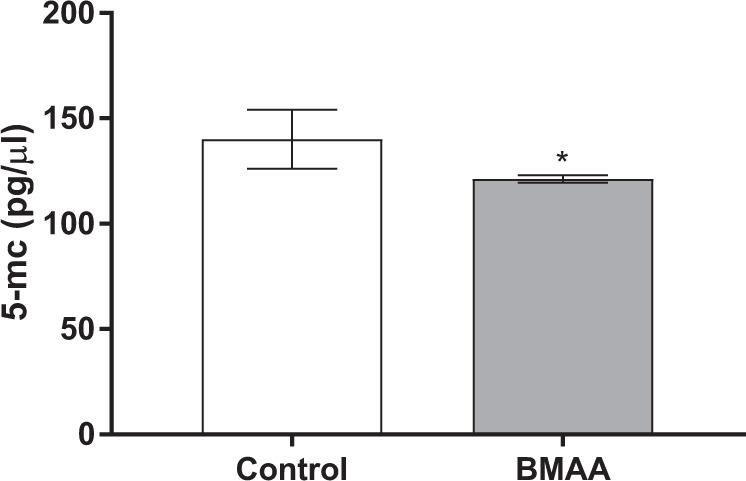
BMAA decreases global DNA methylation in neural stem cells exposed to 100 µM BMAA. DNA methylation was determined by measuring total 5-methylcytosine (5-mc) with ELISA. Values represent mean ± SD from three independent experiments with six replicates each. Statistically significant differences from control are indicated as follows: *p < 0.05 (Student’s *t-*test).

As epigenetic modifications such as DNA methylation play critical roles in the regulation of neural stem cell proliferation and differentiation^27^, we analyzed the global DNA methylation in cells treated with 100 µM BMAA. The results revealed that BMAA decreased global DNA methylation in neural stem cells compared with the control group (Fig. 7).

## Discussion

The development of the nervous system requires an intricate balance between proliferation of the progenitors, differentiation of the correct cell types, and the subsequent migration and connection of these cells. The importance of the regulation of neural development is demonstrated by the many neurodevelopmental disorders that can arise from defects in morphogenesis^34^. In this study, hippocampal neural stem cells were found to be clearly more sensitive to BMAA than hippocampal primary neurons. Exposure to 250 µM BMAA inhibited neural stem cell proliferation through induction of apoptosis, while the non-cytotoxic dose 100 µM BMAA inhibited the differentiation of neural stem cells into neurons, astrocytes and oligodendrocytes. BMAA induced a decrease in cell numbers also in primary neurons, but only at the relatively high concentration 3 mM. This adds additional evidence to previous research showing that BMAA is more neurotoxic for the developing brain^14,15,22,35^ and illustrate the importance to mechanistically elucidate BMAA’s effects during brain development. Importantly, the effects on neural stem cells were mitotically inherited, demonstrating that the BMAA-induced alterations can persist, and even be evident at lower concentrations, in daughter cells after a multitude of cell divisions. In line with this, we observed an altered global DNA methylation pattern in neural stem cells treated with this toxin.

The neural stem cell number is rigorously regulated during central nervous system development. More than half of the immature neurons are deleted in certain brain regions during normal development without interfering with the further development of the remaining cells. Many components of the machinery for programmed cell death are highly expressed in the developing brain, making it more susceptible to unintended activation. Impaired regulation of cell death may lead to brain malformation, impaired learning and memory, or tumor development^36,37^. BMAA exposure induced AIF translocation from mitochondria to the nucleus in hippocampal neural stem cells, which culminated in caspase-independent apoptosis. The mitochondrion-localized flavoprotein AIF translocates to the nucleus where it can bind to DNA and induce DNA fragmentation when apoptosis is induced^38^. Studies have suggested that this protein is important for regulating cell death in multiple neuronal injury pathways^39^, and in particular NMDA-induced neurotoxicity^40^ where the nuclear protein PARP1 induces AIF translocation to the nucleus^41^. As BMAA can induce excitotoxicity via glutamate receptors, it may also induce AIF translocation through PARP1 activation, triggering cell death by apoptosis. Intriguingly, BMAA exposure, at even lower concentration, increased caspase-independent apoptosis also as a mitotically inherited effect in daughter cells.

It is important to point out that the mechanisms by which BMAA affects cell proliferation or triggers cell death can be different depending on the brain area. Recently, we demonstrated that exposure to BMAA reduced striatal neural stem cell proliferation through apoptosis and G2/M arrest^31^. This may be explained by the differences in cell types between striatum and hippocampus. Moreover, the low susceptibility of primary hippocampal neurons to BMAA exposure may be related to the expression of glutamate receptors. Although studies have shown that primary rat neurons grown in culture for 8 days express glutamate receptors, they might not be fully sensitive to glutamate toxicity^42,43^. However, other studies have shown that primary rat neurons are sensitive to glutamate agonists such as quinolinic acid, kainic acid and glutaric acid at this stage of culture^44–46^. It is also possible that different neuronal populations may be more or less sensitive to BMAA exposure, which may explain the variability in the viability of primary neurons in our study.

Neural stem cells give rise to diverse populations of neurons, followed by astrocytes and oligodendrocytes, during embryogenesis^47^. Production of the correct number and subtype of cells during a critical development window is vital for the formation of functional neural circuitry, and defects in this process contribute to neurodevelopmental and neurological disorders^48^. Interestingly, non-cytotoxic BMAA concentrations altered the spontaneous neuronal, astrocytic and oligodendrocytic differentiation in both exposed neural stem cells and daughter cells, demonstrating that the impairments in neural stem cells differentiation can be mitotically inherited to daughter cells after a multitude of cell divisions. The observed effects may be related to previous studies showing that neonatal exposure to BMAA caused cognitive impairments, neuronal degeneration, cell loss, calcium deposits, and astrogliosis in the hippocampus of adult rats^21,24,49^.

The regulation of neurite outgrowth is another crucial process during brain development. Neurons grow out cellular processes, termed neurites, to establish synaptic connections with other neurons, forming highly specialized networks^50^. Abnormal neurite outgrowth and neuronal migration can cause impaired brain functions, where cognitive and motor deficits are the most frequent consequences^51^. Neurons derived from neural stem cells treated with BMAA demonstrated a significantly compromised neurite network even at the lowest concentration tested (50 µM). The effects on the neurite outgrowth also persisted in the daughter cells. Notably, primary neurons treated with 50 µM to 3 mM BMAA did not present any similar alteration.

Epigenetic mechanisms play a critical regulatory role in neural stem cell proliferation and differentiation, and DNA methylation is a key modulator^52^. DNA methylation can influence neural cell identities and altered global DNA methylation often result in cell death during differentiation^53^. Several neurodevelopmental disorders have been linked to proteins involved in the regulation of DNA modification and DNA methylation is closely correlated to stem cell-related diseases^27^. In line with previous studies demonstrating that various toxic agents can induce global DNA hypomethylation^54–56^, BMAA was found to decrease global DNA methylation in neural stem cells. The BMAA-induced effects on neural stem cell proliferation and differentiation, which were mitotically inherited to unexposed cells, could therefore be related to an altered DNA methylation pattern. However, more studies are necessary to identify the specific genes that are hypomethylated and potentially activated by BMAA.

In conclusion, this study provides additional evidence that neural stem cells are more susceptible to BMAA exposure than primary neurons. While BMAA only reduced cell proliferation in primary hippocampal neurons at the highest concentration 3 mM, neural stem cells were clearly affected even at the lowest concentration tested (50 μM). The heritable programming effects induced by BMAA in hippocampal neural stem cells on caspase-independent apoptosis and neural differentiation support the idea that developmental exposures can result in long-lasting brain changes, which may lead to neurodevelopmental disorders or predispose to brain disease later in life.

## Supplementary information

Supplemental Material
