# Supplementary material for: Hippocampal neural stem cells are more susceptible to the neurotoxin BMAA than primary neurons: effects on apoptosis, cellular differentiation, neurite outgrowth, and DNA methylation
Source: Cell Death Dis. 2020 Oct 24;11(10):910. doi: 10.1038/s41419-020-03093-6 (PMC7585576; doi:10.1038/s41419-020-03093-6)
Supplement: Supplementary file 1 — Supplemental Material [file 41419_2020_3093_MOESM1_ESM.docx]

**SUPPLEMENTARY MATERIAL**

**Hippocampal neural stem cells are more susceptible to the environmental neurotoxin BMAA than primary neurons: effects on cellular differentiation, neurite outgrowth, apoptosis and DNA methylation**

Paula Pierozan, Daiane Cattani, Oskar Karlsson

Science for Life Laboratory, Department of Environmental Science, Stockholm University, Stockholm, 114 18, Sweden


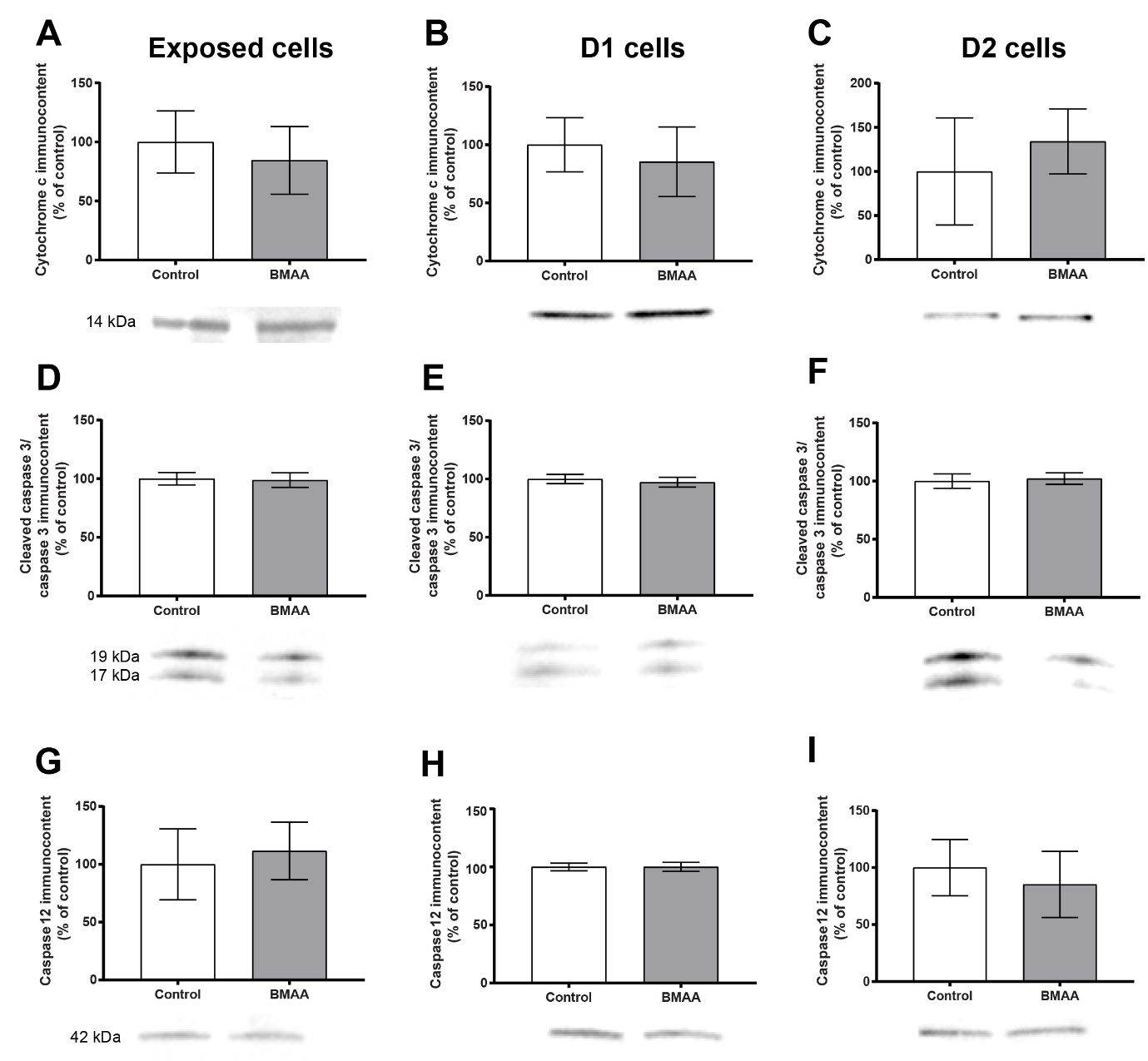


Figure S1: Investigation of cytochrome c (A-C), cleaved caspase 3 (D-F) and caspase 12 (G-I) levels in the cell death triggered by BMAA in neural stem cells. The proteins involved in the mechanisms of apoptosis were studied by western blot in the neural stem cells exposed to 250 µM BMAA (A, D and G) or in the daughter cells, D1 (B, E and H) and D2 (C, F and I), of neural stem cells exposed to 100 µM BMAA. Representative blots of three experiments are shown. β-tubulin was used as a loading control. Values represent mean ± SD from three independent experiments.
